# Application in the analysis of the occlusal force of free-end missing tooth implant restoration with T-SCAN III

**DOI:** 10.3389/fbioe.2023.1039518

**Published:** 2023-04-06

**Authors:** Ming-Le Wu, Peng-Yu Lai, Fan Cheong, Wen-Cheng Zhou, Sang-Hui Xu, Hui Li, Shan Shen

**Affiliations:** ^1^ Department of Stomatology, Affiliated Stomatological Hospital of Jinan University (Daliang Hospital Shunde District Foshan City), Foshan City, Guangdong Province, China; ^2^ School of Stomatology, Jinan University, Guangzhou City, Guangdong Province, China; ^3^ Department of Otorhinolaryngology and Head Neck Surgery, The First Affiliated Hospital of Jinan University, Guangzhou, China; ^4^ Department of Stomatology, The First Affiliated Hospital of Jinan University, Guangzhou, China

**Keywords:** T-SCAN, free-ends, missing implant, occlusal force, stomatology

## Abstract

**Introduction:** The occlusal force of the teeth in the dental arch and the remaining adjacent natural teeth will change after implant restoration with a free-end missing tooth. This study intends to use the T-SCAN III scanner to collect dynamic quantitative data before and after the restoration of free-end implants and to explore the application of the T-SCAN III in redistributing the occlusal force of free-end implants.

**Methods:** In this study, 24 patients with free-end implant restoration were selected, and their occlusion was tested before, immediately after, and 3 months after implant restoration.

**Results:** In all 24 cases, the bite force of the first natural tooth adjacent to the implanted tooth after restoration changed from 19.12% ± 9.48%–12.93% ± 11.47% (*p* < 0.01). For additional data analysis, all cases were further subdivided by single implant and fixed bridge restorations. In 17 cases, there was a successful follow-up after 3 months. The percentage of the total bite force of dental arch with implant increased from 41.92% ± 10.78%–53.06% ± 10.71% (*p* < 0.01).

**Discussion:** This study shows that the free-end implant restoration protects the remaining natural teeth, and the patient’s missing dental arch bite force improves within 3 months of implant restoration.

## 1 Introduction

With the continuous improvement and development of basic research and clinical technology related to dental implants, the success rate of implantation has been increasing annually, reaching over 95%. (Pozzi et al., 2021; Gupta et al., 2022). Dental implant restoration is one of the preferred treatment options for clinicians and patients for restoring missing teeth, free-ends in particular, due to its stability, high patient satisfaction, predictable after-effects, and minimal damage to surrounding tissues (particularly the hard tooth tissue). Occlusion is the basis of masticatory function and an important indicator of the success of oral implant restoration. An ideal bite adjustment is inseparable from adjustment. While adjustment is a basic skill that a stomatologist must master, it is also difficult in dental treatment. The study by Filtchev and Kalachev, (2008) showed that with 32-row teeth, the maximum bite force is distributed on the third molar when the masseter is occluded in the middle; with 28-row teeth, the maximum bite force is distributed on the second molar. From the above research results on the second molars, it can be speculated that the free-end posterior teeth bear a greater biting force. When these teeth are missing, the maximum biting force shifts forward. Studies (Kon et al., 2017) have shown that restoration of free-end posterior teeth can reduce the occlusal load of the remaining natural teeth. Therefore, implant restoration of the free-end missing posterior teeth may protect the adjacent natural teeth.

**FIGURE 1:**
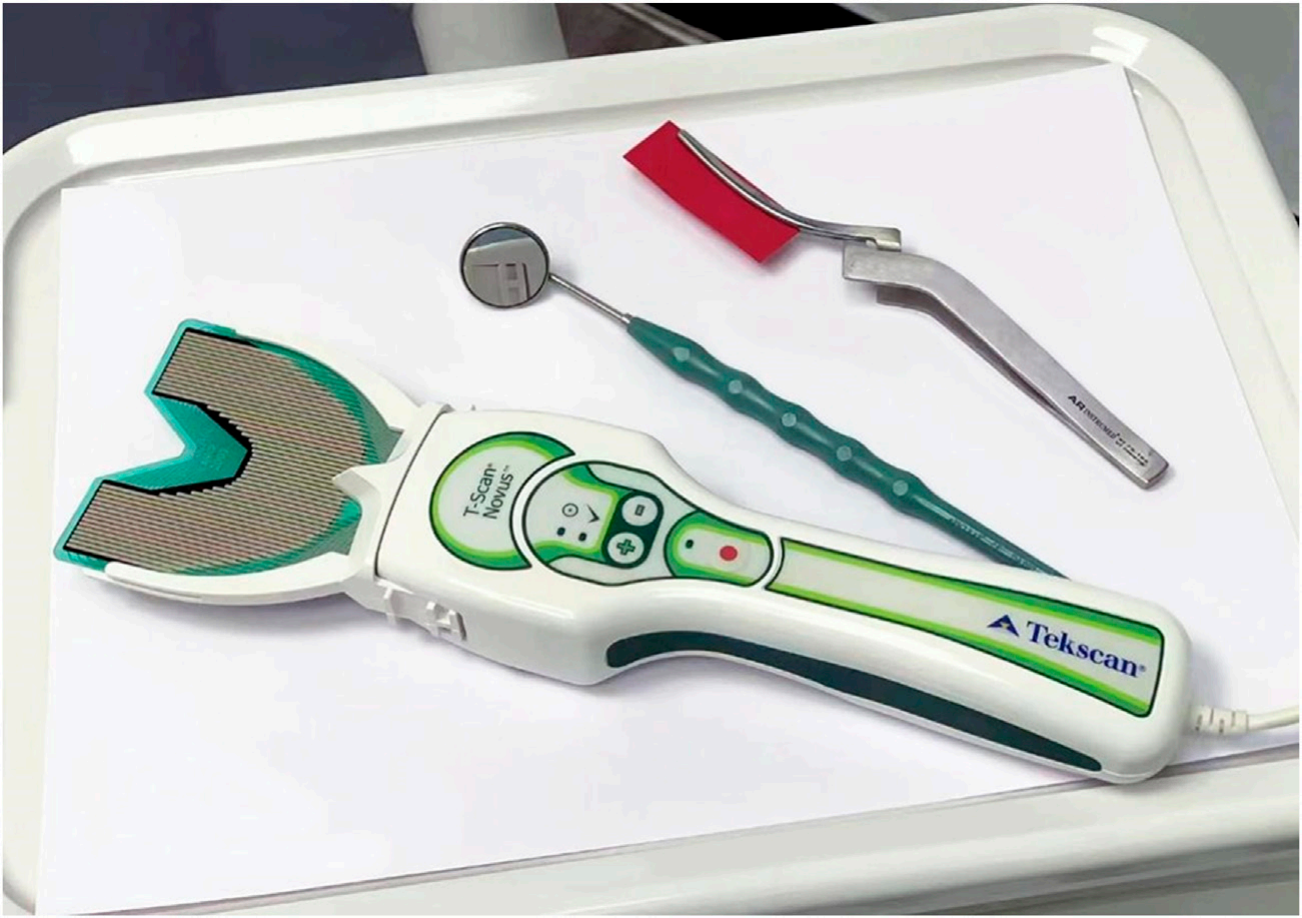
T-SCAN III digital occlusal analysis system.

**FIGURE 2:**
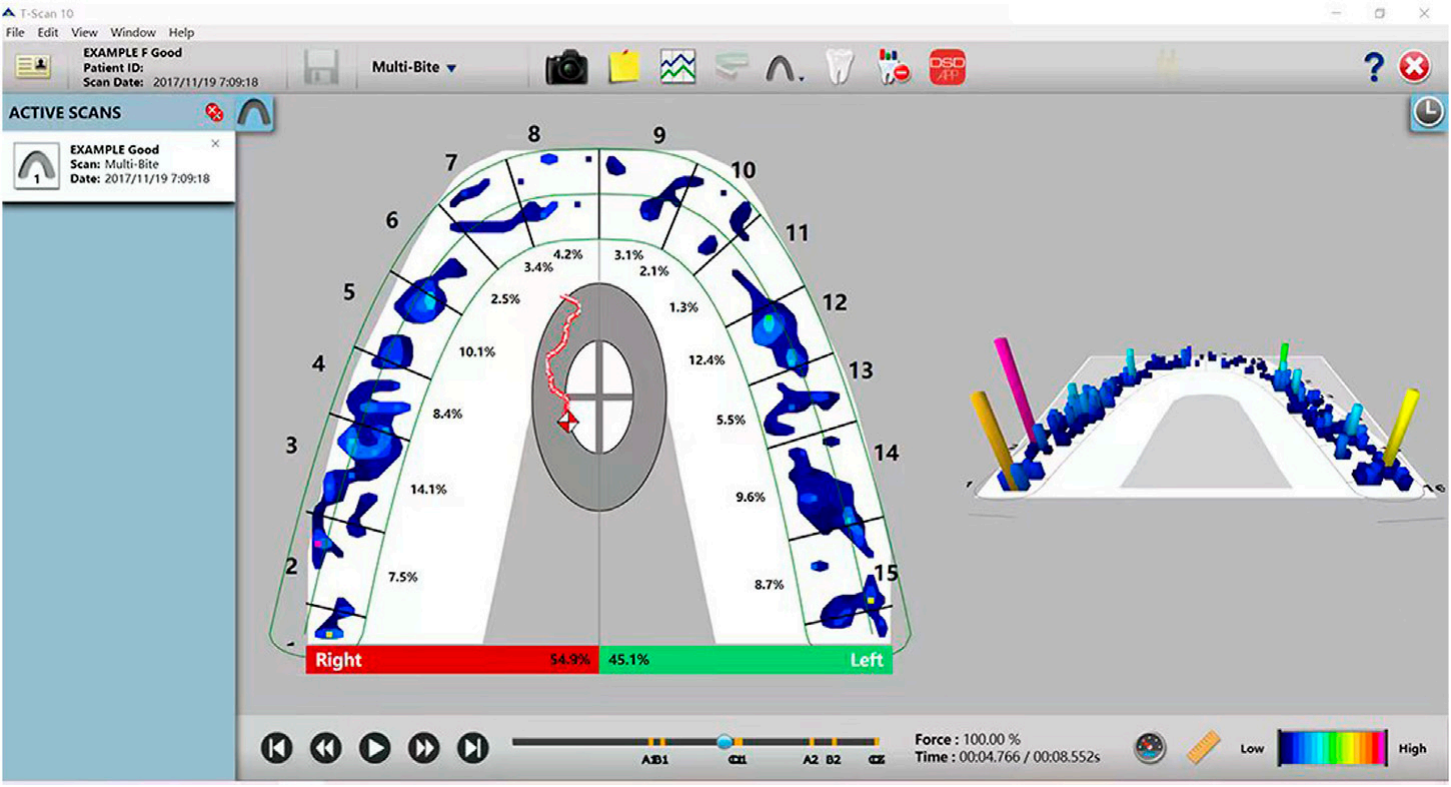
T-SCAN 2D and 3D image information.

The T-SCAN III is an occlusal inspection tool that combines computer technology to quantify occlusal force strength while showing contact location (Figure 1). In 1987, Maness et al. invented the T-SCAN I occlusal recording analyzer (Bozhkova, 2016), which introduced the time parameter for occlusal recording for the first time. They carried out dynamic quantitative analysis of occlusal contact. After 20 years of developing and updating the hardware and software systems, Tekscan invented the T-SCAN III digital occlusal analysis system in 2006. The T-SCAN III sensor measures the total pressure in N/cm^2^ and assigns a percentage of this value to the corresponding area. The recorded data produces a real-time pressure map of occlusal force and distribution over time. This information can then be displayed in 2D and 3D graphics by the Tekscan 10.0 (Figure 2). The software is then instructed to objectively select the frame that applies the maximum total load to the sensor board. Therefore, the system can diagnose and analyze occlusal force changes in patients, accurately identify the distribution and early contact points of an unusually large occlusal force, and accurately guide doctors in performing occlusal adjustments on natural teeth or restorations. This study aims to describe the occlusal force change in patients with free-end implant restoration using the electronic occlusal scanner, T-SCAN III. It also aims to investigate the changes in occlusal force from the initial stage to the period after implant restoration and the changes in the occlusal force of teeth in the dental arch and to determine if free-end implant restoration can protect the adjacent natural teeth.

## 2 Materials and methods

### 2.1 Study sample

The sample consisted of 24 patients who successfully underwent free-end dental implantation at Belong Dental Clinic in Macau between June 20 and January 2022 (12 cases with fixed bridge restoration and 12 with single crown restoration). The patients were aged from 31 to 72, with an average age of 52.79 ± 12.34. The details of gender, age, restoration tooth position, and smoking and drinking habits are shown in Table 1.

**TABLE 1:** Patient information and implant placement.

Gender	Age	Tooth position	Smoke	Quantity	Drinks
M	31	27	Yes	20/day	Occasionally
M	56	16	No	No	Occasionally
M	56	36	No	No	Occasionally
F	53	37	No	No	No
M	67	47	No	No	No
M	35	47	No	No	Occasionally
M	32	47	No	No	No
M	35	17	No	No	No
F	33	47	No	No	No
M	48	37	Occasionally	Occasionally	Occasionally
M	49	34,35,36	No	No	Occasionally
F	58	35,36,37	No	No	No
F	67	36,37	No	No	No
F	47	36,37	No	No	No
F	64	36,37	No	No	No
M	72	45,46,47	No	No	No
	60	14,15,16,17	No	No	No
F	58	36,37	No	No	No
M	70	25,26,27	No	Occasionally	Occasionally
F	58	35,36,37	No	No	No
F	56	36,37	Yes	10/day	Occasionally
F	63	27	No	No	No
M	52	35,36,37	No	No	Occasionally
F	47	17	No	No	No

#### 2.1.1 Inclusion criteria

Patients had to have a unilateral loss of free-end posterior teeth, the right pair of natural teeth, single implant restoration in the rear molars, or fixed restoration of the posterior tooth free-end bridge, and no systemic disease unsuitable for dental implants.

#### 2.1.2 Exclusion criteria

Patients must not have had implant restoration in the opposing jaw, free-end implants requiring bone augmentation, and a history of occlusal trauma.

The Medical Ethics Committee of Jinan University approved this study (approval number: JNUKY-2022–045). All procedures were fully explained to all participants who signed informed consent forms.

### 2.2 Occlusion recording with the T-SCAN III

**FIGURE 3:**
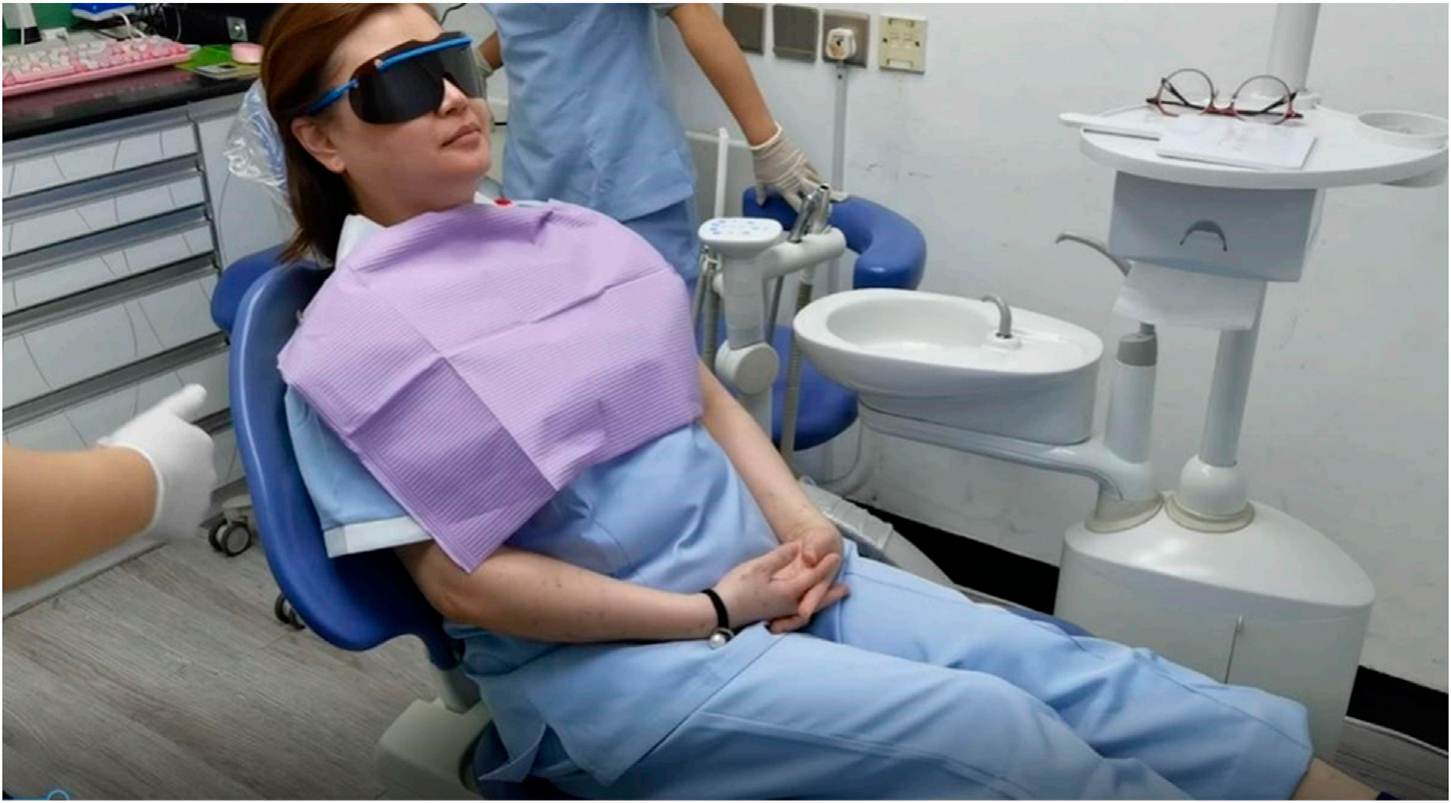
Patient in sitting position.

**FIGURE 4:**
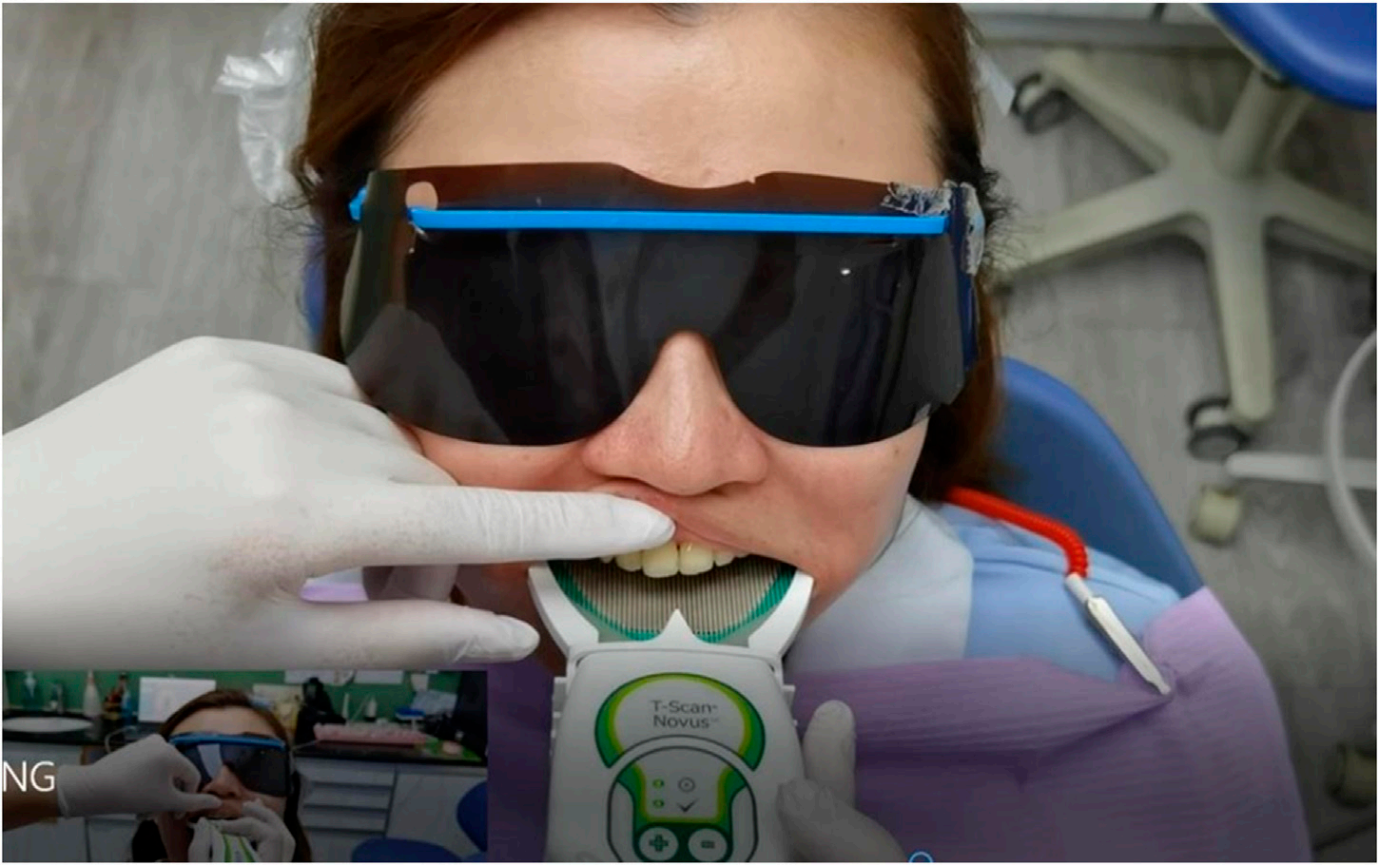
Place handle, align, ask patient to bite at ICP 3 times.

On the day of the restoration, a digital occlusal scan was performed by the physician before and after the restoration using the T-SCAN III. Before the final restoration, the patient was instructed to perform maximum intercuspidal occlusion three times. When the patient could occlude stably, the dental chair was adjusted to a sitting position until the occlusal plane was almost parallel to the ground (Figure 3). The occlusal sensor was then placed in the patient’s mouth and aligned. The handle was then activated to start recording (Figure 4). The patient was then instructed to clench their teeth hard to ensure proper occlusion of the teeth and then asked to release. The patient then repeated this maximal intercuspidal occlusion three times. After the implant restoration was completed, the entire process was repeated, and occlusal adjustment was performed with the articulating paper. The same T-SCAN III handle was used for all measurements in this study. A new sensor sheet was used for each patient to obtain two scans before and after. The collected data was exported to the T-SCAN software, which reported the percentage (%) of the total occlusal pressure (N/cm^2^) at various contact points across the entire dental arch. The exported data was displayed as a sequence of reads over time. The analytical software objectively determined the time point of the maximum occlusal force.

### 2.3 Sample size calculation

The sample size is calculated according to the experimental research method (Zhou et al., 2021), and the analysis index is “the change of occlusal force distribution before and after free end implant restoration”, assuming that the data to be measured is occlusal force, if the standard deviation of the difference in occlusal force of the subjects in this study is For σ = 3 units, every 2 units of occlusal force increase after implant restoration was considered meaningful, α was 0.05, and the test efficiency 1-β was 0.90. The standard deviation of the bite force of the sample is s=(σ = )3 units, δ = 2 units of bite force change, α = 0.05, Zα/2 = 1.96, β = 0.10, Zβ = 1.282.

Calculated as follows:

$$n={\left[\frac{\left(Z_{\alpha /2}+Z_{\beta }\right)S}{\delta }\right]}^{2}$$

The result calculated from the formula is 23.56, that is, at least 24 cases are required for the study.

### 2.4 Statistical analysis

The experimental data were statistically analyzed with IBM SPSS 26.0 software. Measurement data was subject to normal distribution expressed as mean ± standard deviation (x̄ ± s). Measurements such as the changes in dental arches before and after implantation, or contralateral dental arches, were tested by a paired *t*-test, with a testing level of α = 0.05, and *p* < 0.05 was considered statistically significant.

## 3 Results

### 3.1 Occlusal force changes before and after implanted crown placement

Through the records of T-SCAN III, we obtained the original data of the samples (see appendix). We chose the three natural teeth adjacent to the implant as the study object because we found that the occlusal force variation of the fourth and above natural teeth was almost zero. Occlusal force data from the 24 cases were analyzed before and after repair. Before repair measurements were 41.21% ± 15.92%, and after repair were 42.99% ± 14.10%, without a significant change from before and after repair (*p* > 0.05). Comparing the three adjacent natural teeth before and after restoration, it was found that the occlusal force of the first adjacent natural teeth decreased from 19.12% ± 9.48% before restoration to 12.93% ± 11.47% after restoration (*p* < 0.05) (Table 2).

**TABLE 2:** Changes of occlusal force before and after dental implant restoration.

	Before (*N* = 24)	After (*N* = 24)	*t*	*P*
**Arch with implant,%**	41.21 ± 15.92	42.99 ± 14.10	−0.619	0.542
**1** ^**st**^ **adjacent tooth,%**	19.12 ± 9.48	12.93 ± 11.47	3.284	0.003**
**2** ^**nd**^ **adjacent tooth,%**	8.41 ± 5.97	7.63 ± 8.65	0.402	0.691
**3** ^**rd**^ **adjacent tooth,%**	6.65 ± 7.16	5.11 ± 6.63	1.567	0.131
**Contralateral dental arch,%**	58.79 ± 15.92	57.01 ± 14.10	0.619	0.542

*p* < 0.01 *p* < 0.05.

### 3.2 Occlusal force changes before and after free-end single implant and bridge implant dental restorations

After tooth restoration and implant adjustment based on functional occlusal protection, the occlusal force of the dental arch increases and decreases, and careful analysis shows that the number of functioning teeth units may factor into this result. (Latimer et al., 2021). In the occlusal force distribution, the number of functional teeth units (FTUs) also influences the overall occlusal force of the dental arch. (van der Bilt, 2011). Due to the apparent difference in the number of functional teeth added in the fixed bridge restoration, the 24 cases were further subdivided into a single implant or fixed bridge restoration group, and the data were analyzed. The influence on the overall force distribution was also discussed.

Twelve patients aged 30–67 years and an average age of (46.27 ± 13.47) underwent a single-implant restoration of the posterior extension. This group included four women and eight men. Statistical analysis of these 12 cases showed that the occlusal force decreased from 50.95% ± 10.35% before implant restoration to 41.65% ± 9.97% after restoration (*p* < 0.01). The occlusal force at the implant position increased from 1.73% ± 2.80%–6.44% ± 7.38% (*p* < 0.05). In addition, the occlusal force of the three natural teeth adjacent to the implant was also analyzed and compared. The occlusal force of the first adjacent natural tooth decreased from 22.88% ± 7.15%–14.33% ± 10.25% (*p* < 0.05) and dropped from 9.44% ± 5.98%–6.12% ± 3.33% (*p* < 0.05) for the adjacent second natural tooth, which was statistically significant (Table 3).

**TABLE 3:** Single implant restoration occlusal force change.

	Before (*N* = 12)	After (*N* = 12)	*t*	*P*
**Arch with implant,%**	50.95 ± 10.35	41.65 ± 9.97	4.267	0.001**
**Implant,%**	1.73 ± 2.80	6.44 ± 7.38	−2.323	0.040*
**1** ^**st**^ **adjacent teeth,%**	22.88 ± 7.15	14.33 ± 10.25	3.108	0.010*
**2** ^**nd**^ **adjacent teeth,%**	9.44 ± 5.98	6.12 ± 3.33	2.761	0.019*
**3** ^**rd**^ **adjacent teeth,%**	9.11 ± 6.08	6.68 ± 5.50	1.345	0.206
**Contralateral dental arch,%**	49.05 ± 10.35	58.35 ± 9.97	−4.267	0.001**

*p* < 0.01 *p < 0.05.

The overall occlusal force of the implant-containing arch was reduced after single implant restoration. Careful analysis of each tooth position shows that the occlusal force of the implanted tooth increases, but the occlusal force of the adjacent natural teeth has decreased. Due to the decrease in the occlusal force of the adjacent natural teeth, the overall occlusal force percentage of the implanted dental arch was also reduced. The rate of occlusal force against the teeth in the ipsilateral dental arch was reduced after implant placement. However, the occlusal force of adjacent natural teeth showed a downward trend.

The remaining 12 patients underwent fixed-bridge implant repair, were aged 47–72 years, with an average age of 59.25 ± 7.89, and included four men and eight women. In contrast to single implant restoration, the occlusal rate force of the dental arch in patients with fixed-bridge restoration increased from 31.47% ± 14.68% before restoration to 44.33% ± 17.68% after restoration (*p* < 0.01) (Table 4).

**TABLE 4:** Occlusal force change before and after implant bridge restoration.

	Before (*N* = 12)	After *(N* = 12)	*t*	*P*
**Arch with implant,%**	31.47 ± 14.68	44.33 ± 17.68	−4.671	0.001**
**1** ^**st**^ **adjacent teeth,%**	15.37 ± 10.30	11.53 ± 12.88	1.529	0.154
**2** ^**nd**^ **adjacent teeth,%**	7.38 ± 6.04	15.15 ± 23.61	−1.216	0.249
**3** ^**rd**^ **adjacent teeth,%**	4.19 ± 7.55	3.54 ± 7.51	0.817	0.431
**Contralateral dental arch,%**	68.53 ± 14.68	55.67 ± 17.68	4.671	0.001**

*p* < 0.01.

In more than two implant restorations, completely different results could be observed from single implant restorations, with a statistically significant increase in the occlusal force rate in the implanted dental arch. When analyzing this result, it was found that it may be because there are more missing teeth in the implant bridge restoration with a smaller number of FTUs before restoration. The subsequent increase in the FTU after restoration helps the teeth involved in the occlusion function better. This increase in FTUs leads to a corresponding rate increase in the occlusal force, resulting in the vastly different results from an extension with a single free-end implant restoration. Occlusal contact and the occlusal area of natural teeth and the number of posterior teeth are positively correlated with masticatory performance and efficiency, the occlusal force of maximal molars, and maximal levator mandibular mobility. (Bakke et al., 1990; Wilding, 1993; Goshima et al., 2010). In the free-end implant bridge restorations, a significant increase in the number of teeth resulted in a substantial rate increase in the occlusal force of the dental arch.

### 3.3 Comparison of the bite force immediately after restoration and at 3 months

Of the 24 cases, 17 were successfully followed up for 3 months. The other seven were not included in the follow-up criteria because the follow-up time was shorter than 3 months, or other dental restorations were performed during the process. For these 17 follow-up cases, three intercuspidal occlusions were also performed, recorded with the T-SCAN III, and the data were statistically analyzed. Among the 17 cases in the return visit, 10 males and seven females had an average age of 51.59 ± 12.86. Ten were repaired with single crowns, and the other seven were repaired with implant-fixed bridge repairs.

During follow-up, the occlusal force rate of the implanted dental arch increased from 41.92% ± 10.78% immediately after restoration to 53.06% ± 10.71% (*p* < 0.01). At the same time, the occlusal force of the contralateral dental arch also decreased, and the occlusal force of the implanted dental arch increased after 3 months in these 17 patients, whether it was a single crown restoration or a fixed restoration bridge restoration (Table 5). In addition, the occlusal force of the three adjacent natural teeth also increased during the follow-up visit. Still, the data were not statistically significant, which may be related to the insufficient sample size.

**TABLE 5:** Occlusal force information after restoration and after 3 months.

Variable	Immediately (N = 17)	Three months later (N = 17)	*T*	*P*
**1** ^**st**^ **adjacent teeth, %**	58.08 ± 10.78	46.72 ± 10.52	4.373	<0.001**
**2** ^**nd**^ **adjacent teeth, %**	41.92 ± 10.78	53.06 ± 10.71	−4.210	<0.001**
**3** ^**rd**^ **adjacent teeth, %**	10.20 ± 8.10	12.55 ± 8.38	−1.823	0.087
**Arch with implant, %**	7.34 ± 10.21	8.53 ± 10.47	0.197	0.394
**Contralateral dental arch, %**	4.46 ± 6.13	11.78 ± 32.62	0.160	0.319

The follow-up results showed that whether it was a single implant or a fixed restoration bridge restoration, the occlusal force of the fix pertial denture patients in the implant area increased significantly under the guide of occlusal habits and a 3-month adaptation time. Compared with the restoration of a single free-end dental implant, a patient with the fixed restoration bridge implant restoration can achieve the average occlusal force on both sides after 3 months. The occlusal relationship, temporomandibular joint (TMJ), and overall oral health are good phenomena that clinicians can use as a reference when repairing extended-end implants for patients.

### 3.4 Changes in the occlusal force of both dental arches before and after restoration and after 3 months

Twenty patients were selected who had no missing teeth, no unilateral chewing habits, and no restorations and the occlusal force of the left and right dental arches was compared. It was found that the difference in the bilateral occlusal force was not statistically significant (*p* > 0.05), indicating that in the normal control group, the occlusal force on both sides was relatively average. A comparison of the occlusal force distribution of the bilateral dental arches of the patients before and after the restoration of the edentulous extension and the 3-month follow-up was conducted. The occlusal force of the arch was 41.21% ± 15.92% lower than the contralateral dental arch occlusal force of 58.79% ± 15.92% (*p* < 0.001). After tooth placement, the occlusal force of the implant arch was 42.99% ± 14.10%, which was also lower than the contralateral arch occlusal force of 57.01% ± 14.10% (*p* = 0.001). At the 3-month follow-up, it was found that after the patient’s occlusal guidance and adaptation, there was no difference in the rate of occlusal force of the patient’s bilateral dental arches (*p* > 0.05), indicating that the patient’s bilateral dental arch occlusion had improved after 3 months (Table 6). The force distribution showed improvement and was closer to the normal control group.

**TABLE 6:** Comparison of the occlusal force of bilateral dental arches before, after and 3 months after restoration.

	Total	Arch with implant,%	Contralateral dental arch,%	*t*	*P*
**Before crown**	50.00 ± 18.08	41.21 ± 15.92	58.79 ± 15.92	−3.827	<0.001**
**After crown**	50.00 ± 15.65	42.99 ± 14.10	57.01 ± 14.10	−3.443	0.001**
**3 months later**	49.89 ± 10.94	53.06 ± 10.71	46.72 ± 10.52	1.742	0.091

## 4 Discussion

The occlusion of the teeth, TMJ, masticatory muscles, ligaments, and related tissues constitute the masticatory system. The occlusion of the teeth is also affected by factors such as the arrangement of the teeth, the size of the dental arch, the position of the teeth, and the order of the teeth eruption. (Chutchalermpan et al., 2018). A normal occlusion is very important for coordinating the masticatory system. Traditional occlusal examination methods have indirect implications for occlusal force. T-SCAN has been shown to overcome this limitation. The study by Bernd Koos et al. (Koos et al., 2010) pointed out that the occlusal measurement method of T-SCAN is superior to traditional occlusal inspection methods, especially the force analysis of each tooth. Trpevska et al, (2014) also indicated that the T-SCAN system accurately identified tooth contact. The traditional occlusal adjustment process matched the patient’s subjective feelings in the study by Ruttitivapanich et al, (2019) and improved doctor–patient communication while minimizing complications during post-repair follow-up or occlusal imbalance, providing a more predictable treatment outcome. (Afrashtehfar and Qadeer, 2016). It has also been proposed that a T-SCAN is a consistent and reproducible method for occlusal examination. (Floriani et al., 2022). As a cutting-edge biotechnology, T-SCAN plays a vital role in the accelerated transition to digitalization in the field of dentistry. T-SCAN is a mature technology of more than 35 years. It has been updated and iterated since it was born in 1981. T scan I, T scan II, T scan III, T scan III (software 5,6,7), T scan with turbo mode recordings, T scan 8, T scan 9, and now has been updated to T scan 10 (Bathiya and Pisulkar, 2020). It can perform three-dimensional evaluation of the occlusal situation, can evaluate the relative occlusal force and the timing of occlusal contact, and can record work without saliva interference. T-SCAN can calculate the force center of the dental arch and evaluate the upper and lower arches in the front and rear quadrants. There is no doubt that T-SCAN can provide professional training for dentists and improve the accuracy of diagnosis and treatment. Nowadays, medical legal issues are becoming more and more prominent, and the validity of T-SCAN medical legal documents is the highest (Pyakurel et al., 2013).Dental implants are usually directly osseointegrated with the bone, eliminating the space for physiological movement. Compared to natural teeth, implants can only move 10–50 mm horizontally and 3–5 mm in the axial direction. Therefore, although the tooth can accommodate movement by sinking or slightly rotating, the occlusal forces are concentrated at the bone level around the top of the implant, and the implant–bone interface absorbs all forces, (Terauchi et al., 2015; Sheridan et al., 2016), which may lead to implantation body bone resorption. Due to the different combinations of dental implants and natural teeth, to reduce bone resorption caused by excessive occlusal force, it is recommended to focus on heavy occlusion and light contact in the occlusal restoration of dental implants. (Sheridan et al., 2016; Luo et al., 2019). Traditional occlusal adjustment methods cannot quantify the magnitude of the occlusal force and the occlusion order. To avoid subjectivity in interpreting occlusal articulating paper marks, dental research introduced the T-SCAN computerized occlusal analysis system.

The effect of differences in the occlusal contact area of mandibular free-end edentulous implants on the periodontal mechanical sensitivity threshold of adjacent premolars was investigated in the article by Terauchi et al, (2015) In their study, it was shown that there is a correlation between the occlusal area of mandibular extension implants and the periodontal mechanical sensitivity threshold of adjacent natural teeth and that the occlusal balance between implants and the remaining teeth is critical. An implanted occlusal area that is too small may inflict a burden on the periodontal threshold of the adjacent natural teeth. In contrast, one that is too large will cause damage to the implant. Therefore, protection is critical. In this study, the T-SCAN III was used to investigate the redistribution of the occlusal force of each tooth after free-end implant restoration. The results showed that after free-end implant restoration, the occlusal force of the implant increased, while the adjacent natural occlusal force of the teeth was reduced. Combined with the research of Rie Terauchi et al., the occlusal force distribution after restoration shows that the free-end implant has a particularly protective effect on the adjacent natural teeth.

Van Der Bilt, in a review on assessing the effect of mastication on oral rehabilitation, (van der Bilt, 2011), states that the number of FTU and occlusal force are determinants of masticatory performance. In contrast, occlusal force depends on the number of FTUs involved. Therefore, at the free-end implant bridge restoration, the increased occlusal force of FTU was greater than the decreased occlusal force of the adjacent natural teeth. The results differed from the occlusal force change of free-end single implants. In addition, Miguel A. Roque et al, (2017) also found that for a single implant tooth with intermediate missing teeth, the overall occlusal force of the dental arch was increased and the occlusal force acting on the remaining natural teeth was raised due to the increase of FTU, which was similar to the results of this study. Appropriate occlusal force is beneficial long-term to the implant and the natural teeth. Some literature (Greenstein et al., 2016; Latimer et al., 2021) points out that excessive occlusal force will cause mesial movement of natural teeth, resulting in gaps between implants and natural teeth, eventually leading to issues such as peri-implantitis. Therefore, the occlusal force distribution must be carefully and accurately analyzed and adjusted after restoration. In the analysis of the occlusal force of the adjacent natural teeth, the trend of occlusal force decreased, and it was found that the data for the first adjacent natural tooth was statistically significant. This indicates that the free-end implants have a particularly protective effect on the adjacent natural teeth.

A study (Goshima et al., 2010) has shown that in single dental implant restoration, the implant restoration positively affects the subjective perception and quality of life of patients with missing teeth due to increased occlusal contact area, occlusal force, and masticatory performance after 1 month of restoration. Kim et al, (2021) compared the initial changes of occlusal force and occlusal contact after the restoration of single molar implants and the data immediately after 1 month. They showed that the occlusal force 1 month after implantation was significantly increased compared to before implantation. In addition, there are clinical studies (Zhang et al., 2021) on changes in occlusal contact time and occlusal force of individual implants, investigating at intervals of half a month, 3 months, 6 months, and a year after implant restoration. The results showed that the percentage of occlusal force changed most significantly 3 months after repair, and the occlusal force continued to increase with time. (Ding et al., 2021). Previous studies have also found that the occlusal force after implant restoration rises with time, (Ding et al., 2021), and the order of tooth contact in the occlusion is particularly important for continuous tracking of the occlusal force of free-end implants. Therefore, in the future, the long-term effect of free-end implant restoration can be followed by a long-term follow-up to check whether the occlusal force will increase with time. A comparative study can also be conducted on the long-term stability and bone resorption of implants. This could provide a more objective basis for the need for regular adjustment in the occlusal force of dental implants.

A limitation of this study is that the influence of implant design features on the experimental results was not fully discussed. Due to the specific situation of each patient, the diameter and length of the implants we use are different. Studies have shown that implant material, implant diameter, implant length, implant-abutment connection type, bone density and loading conditions all affect the biomechanical behavior of implants and abutments in terms of stress distribution and bone strain distribution (Pozzi et al., 2021; Gupta et al., 2022). Kim et al. (2020) found through research that under the same load conditions, the stress value of tissue level implants at the implant-abutment junction is lower than that of bone level implants. In addition, tissue-level implants have the advantages of less microgap formation and longer fatigue life compared to bone-level implants (Lee et al., 2021). In terms of the choice of implant-abutment connection type, the two most common configurations are hexagonal and non-hexagonal abutments, and the hexagonal configuration was found to increase the stability of implants and abutments more than the non-hexagonal configuration. Furthermore, although its effect is not as pronounced as that of the abutment type, a large crown-to-implant ratio (CIR) increases bone strain and stress in the implant component, especially under oblique loading (Lee et al., 2020). Therefore, in the future, patients with the same or similar implant design characteristics should be selected for research, or efforts should be made to eliminate or reduce the impact of this variable on the study, so as to make the study more valuable.

## 5 Conclusion

Through this study, we found that extension implants have a protective effect on adjacent natural teeth within the same arch. After restoration of free-end implants, the occlusal force of the implanted teeth was increased, while that of the adjacent natural teeth was relieved. Through subsequent follow-up, we found that the patient’s missing tooth arch achieved occlusal force redistribution 3 months after the implant restoration. As a tool for occlusal diagnosis, T-SCAN III occlusal force analyzer can provide clinicians with more intuitive inspection basis. Dentists can use T-SCAN III in conjunction with traditional occlusal adjustment methods to perform more accurate tooth adjustments and reduce occlusal adjustment errors caused by subjective factors.

## Data Availability

The original contributions presented in the study are included in the article/supplementary material, further inquiries can be directed to the corresponding authors.
